# Primary hyperparathyroidism presenting with pathological fractures mimicking malignancy: a case from Tanzania

**DOI:** 10.1093/omcr/omag023

**Published:** 2026-03-23

**Authors:** Noorein Omar, Lutfi Abdallah, Mahmoud Abeid, Tareeq Saleh, Mudhihir Rupia, Hilda Makungu, Sibtain Moledina

**Affiliations:** Department of Internal Medicine, Muhimbili University of Health and Allied Sciences, United Nations Road, Upanga West, Ilala District, Dar es Salaam, P.O. Box 65001, Tanzania; Department of Internal Medicine, Muhimbili University of Health and Allied Sciences, United Nations Road, Upanga West, Ilala District, Dar es Salaam, P.O. Box 65001, Tanzania; Department of Internal Medicine, Muhimbili National Hospital, United Nations Road, Upanga West, Ilala District, Dar es Salaam, P.O. Box 65000, Tanzania; Department of Internal Medicine, Muhimbili University of Health and Allied Sciences, United Nations Road, Upanga West, Ilala District, Dar es Salaam, P.O. Box 65001, Tanzania; Department of Internal Medicine, Muhimbili University of Health and Allied Sciences, United Nations Road, Upanga West, Ilala District, Dar es Salaam, P.O. Box 65001, Tanzania; Department of Internal Medicine, Muhimbili University of Health and Allied Sciences, United Nations Road, Upanga West, Ilala District, Dar es Salaam, P.O. Box 65001, Tanzania; Department of Radiology, Muhimbili National Hospital, United Nations Road, Upanga West, Ilala District, Dar es Salaam, P.O. Box 65000, Tanzania; Department of Internal Medicine, Muhimbili University of Health and Allied Sciences, United Nations Road, Upanga West, Ilala District, Dar es Salaam, P.O. Box 65001, Tanzania

**Keywords:** primary hyperparathyroidism, pathological fractures, hypercalcemia, parathyroidectomy, hungry bone syndrome, Tanzania

## Abstract

Primary hyperparathyroidism (PHPT) is commonly detected incidentally in high-income settings, but in resource-limited contexts it may still present with advanced skeletal disease. We report a 45-year-old woman with a seven-year history of progressive bone pain culminating in pathological fractures of the femoral neck and proximal humerus after minor trauma. Malignancy and myeloma were excluded by histology and hematologic work-up. Biochemistry showed severe hypercalcemia with strikingly elevated parathyroid hormone and alkaline phosphatase; imaging demonstrated diffuse osteopenia and localized enlarged parathyroid glands. She underwent parathyroidectomy with subsequent hungry bone syndrome, managed with calcium and vitamin D supplementation, followed by functional recovery and planned orthopedic fixation. This case highlights the risk of misdiagnosis where routine biochemical screening is limited, and emphasizes early consideration of metabolic bone disease in patients with unexplained fractures once malignancy is excluded.

## Introduction

Primary hyperparathyroidism (PHPT) is a common endocrine disorder characterized by excess parathyroid hormone secretion [1]. In high-income countries, most cases are detected incidentally during biochemical screening, and overt skeletal disease is rare [2]. In contrast, delayed recognition in resource-limited settings may result in pathological fractures and severe disability [3]. Rare manifestations such as brown tumors can mimic metastatic bone disease, complicating diagnosis [4]. This report describes a Tanzanian woman with longstanding bone pain and multiple fragility fractures initially suspected to be malignant, ultimately diagnosed as PHPT.

## Case report

A 45-year-old Tanzanian woman presented with severe bone pain and inability to mobilize after minor-trauma fractures of the left femoral neck and right proximal humerus. Her symptoms began seven years earlier as persistent low back pain attributed to lumbar spondylosis, managed conservatively without improvement. Internal fixation failed because of extreme bone fragility, and malignancy was suspected.

On examination, she appeared pale, afebrile, and hemodynamically stable. Examination demonstrated tenderness over the left thigh and right upper arm, with obvious deformity and limited range of motion at the hip and shoulder. There was no lymphadenopathy, organomegaly, or systemic features of malignancy.

**Figure 1:**
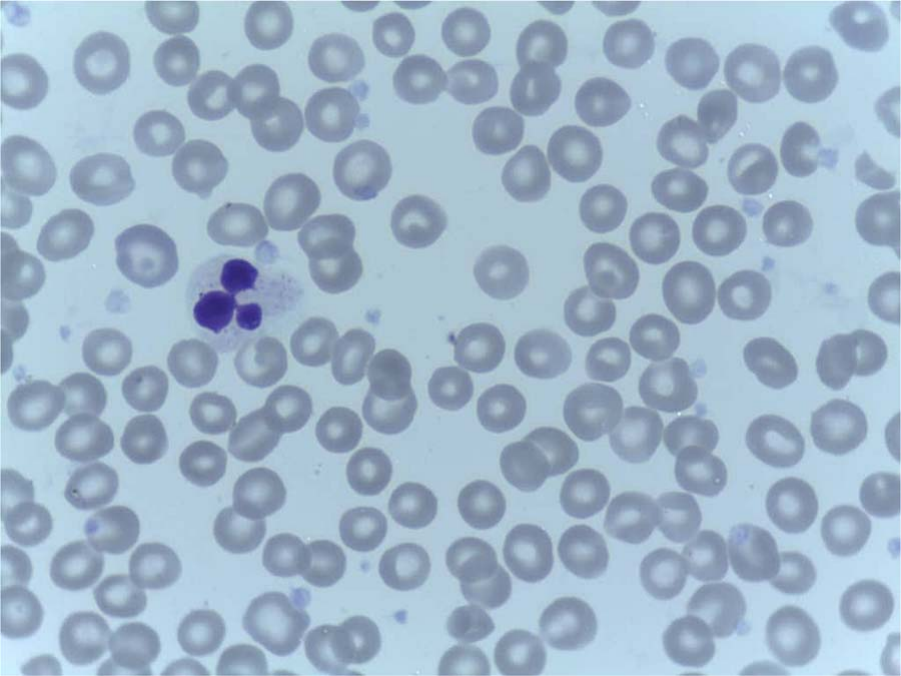
Peripheral blood smear showing normocytic, normochromic anemia with adequate platelets; no rouleaux formation.

**Figure 2:**
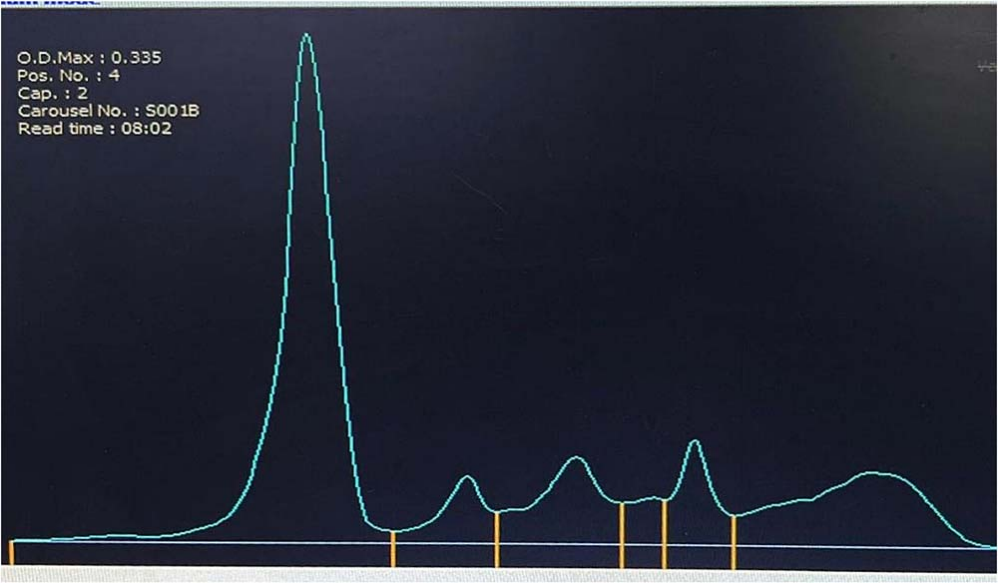
Serum electrophoresis with normal distribution of protein fractions; no monoclonal spike.

**Figure 3:**
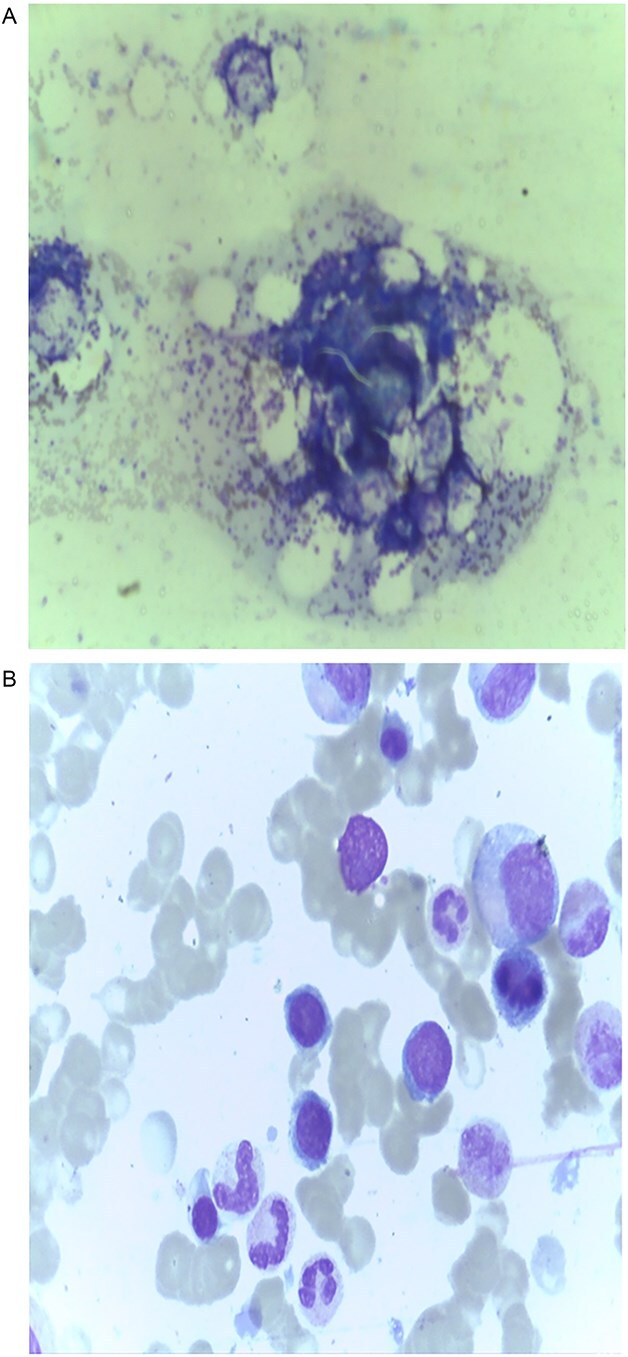
Bone marrow aspiration and biopsy showing normal morphology and cellularity; no increase in plasma cells. (A) Bone marrow aspirate showing normocellular marrow with preserved trilineage hematopoiesis. (B) Trephine biopsy from the fracture site demonstrating normal architecture with no evidence of plasma cell dyscrasia or metastatic infiltration.

**Figure 4:**
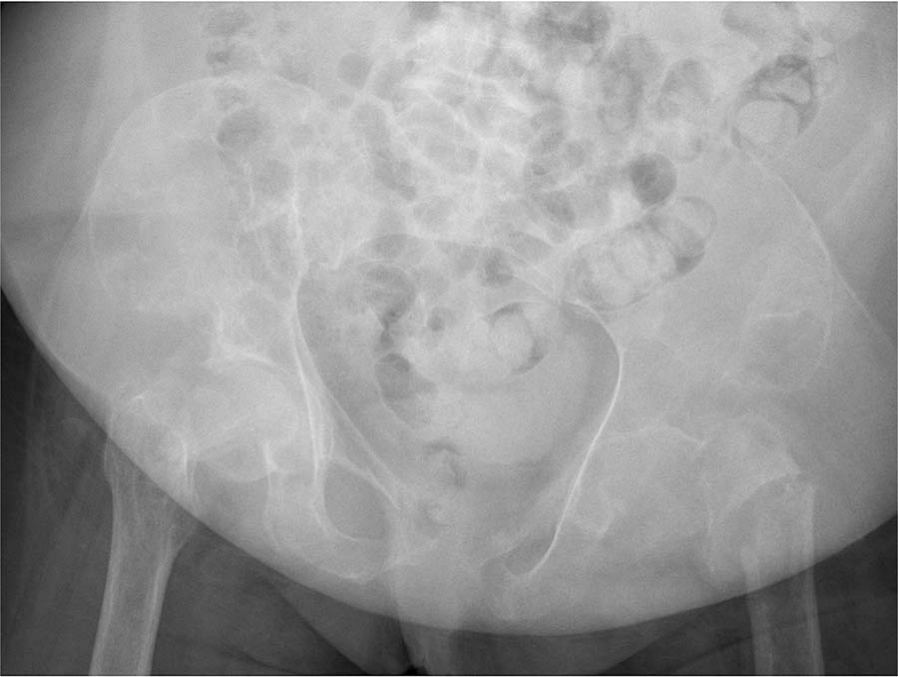
Pelvic x-ray coronal view, linear lucency through the left femoral neck with associated cortical irregularity and focal discontinuity of the medial and lateral cortex concerning for pathological fracture.

**Figure 5:**
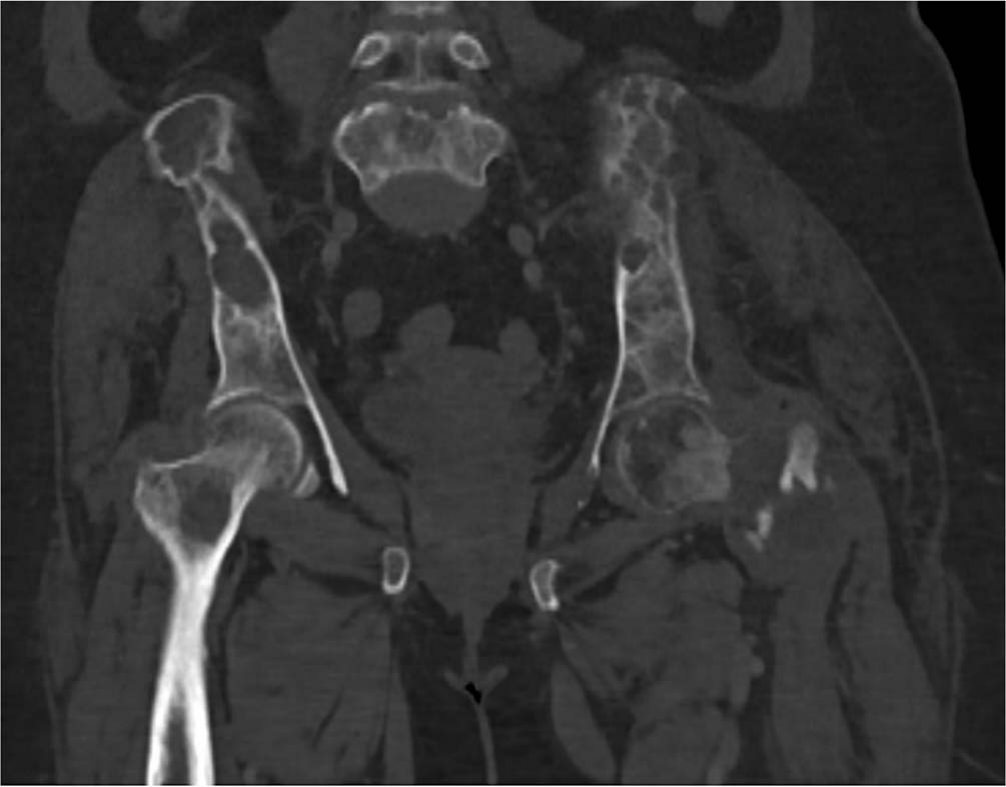
Pelvis CT scan in coronal view, bone window showing diffuse osteopenia of the sacrum and iliac bones with cortical thinning and multiple pathological fractures of the left femoral neck, including transcervical and intertrochanteric components.

**Figure 6:**
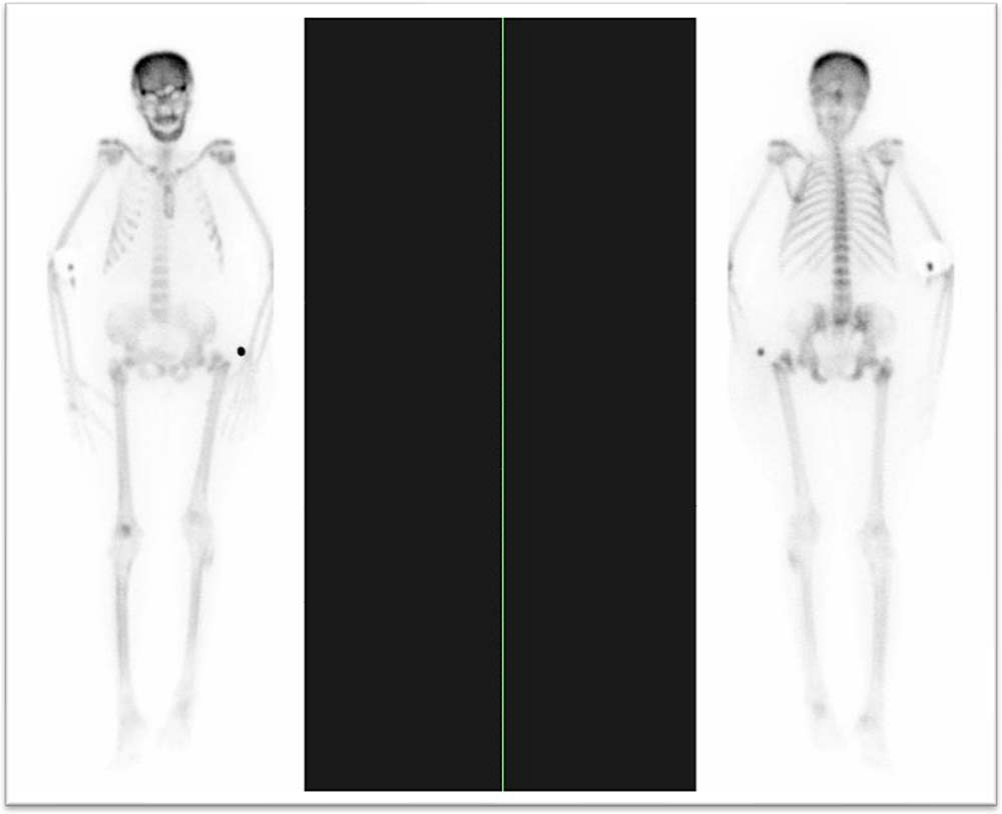
Bone scan demonstrating diffuse uptake compatible with metabolic bone disease; no focal malignant uptake. (A)whole-body bone scintigraphy demonstrating diffuse increased uptake throughout the axial and appendicular skeleton, consistent with metabolic bone disease. (B)No focal areas of abnormal tracer accumulation suggestive of malignant deposits.

**Figure 7:**
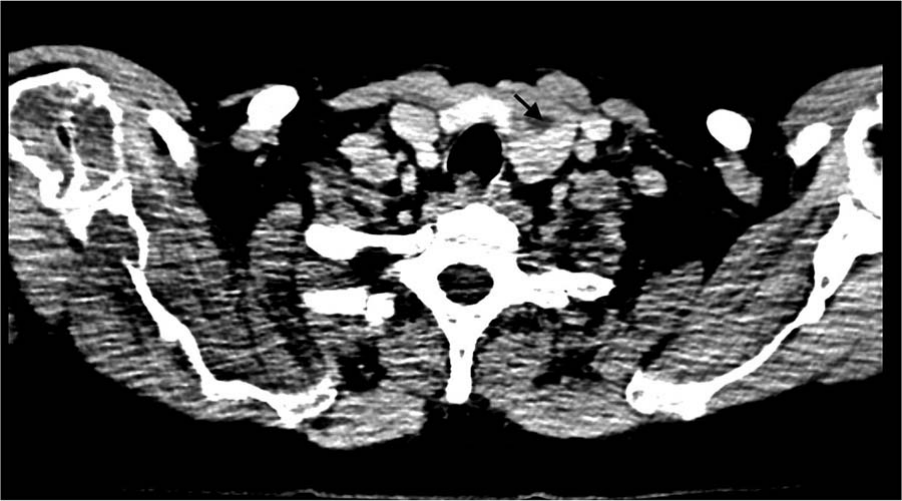
Neck CT (post-contrast, axial) localizing enlarged parathyroid glands. A 2.45 × 2.42 × 3.3 cm heterogeneously enhancing mass with central necrosis is seen in the postero-inferior aspect of the left thyroid lobe. The mass abuts the ipsilateral thyroid lobe and trachea without evidence of invasion. Carotid vessels and the esophagus are intact.

The patient underwent an extensive series of investigations to delineate the cause of her progressive bone fragility and to rule out malignancy. Initial laboratory evaluation (Table 1) demonstrated marked hypercalcemia, low serum phosphate, and significantly elevated alkaline phosphatase, suggestive of high bone turnover. Parathyroid hormone was inappropriately elevated, pointing towards primary hyperparathyroidism. Kidney function was preserved, and vitamin D was relatively insufficient. Peripheral blood smear showed a normocytic normochromic anemia without rouleaux (Fig. 1). Myeloma screening was negative, with no monoclonal proteins detected, and both bone marrow and fracture-site biopsy excluded plasma cell dyscrasia or metastatic disease (Figs. 2 and 3). Radiographs demonstrated a linear lucency through the left femoral neck with associated cortical irregularity and focal discontinuity of the medial and lateral cortex concerning a pathological fracture (Fig. 4). CT pelvis in coronal bone-window view showed diffuse osteopenia of the sacrum and iliac bones, accompanied by cortical thinning and multiple pathological fractures of the left femoral neck, including both transcervical and intertrochanteric components (Fig. 5). Bone scintigraphy demonstrated diffuse uptake compatible with metabolic bone disease, and neck imaging localized enlarged parathyroid glands (Figs. 6 and 7).

**Table 1:** Laboratory results showing marked hypercalcemia, low–normal phosphorus, markedly elevated PTH and ALP indicating high bone turnover, and vitamin D within reference range.

Test	Result(s)	Reference range
Calcium	3.25–3.81 mmol/L	2.2–2.7 mmol/L
Phosphorus	0.7–1.0 mmol/L	0.7–1.5 mmol/L
PTH	2300 pg/mL	0–55 pg/mL
ALP	1212 U/L	6–80 U/L
Vitamin D	32.8 ng/mL	20–40 ng/mL

**Figure 8:**
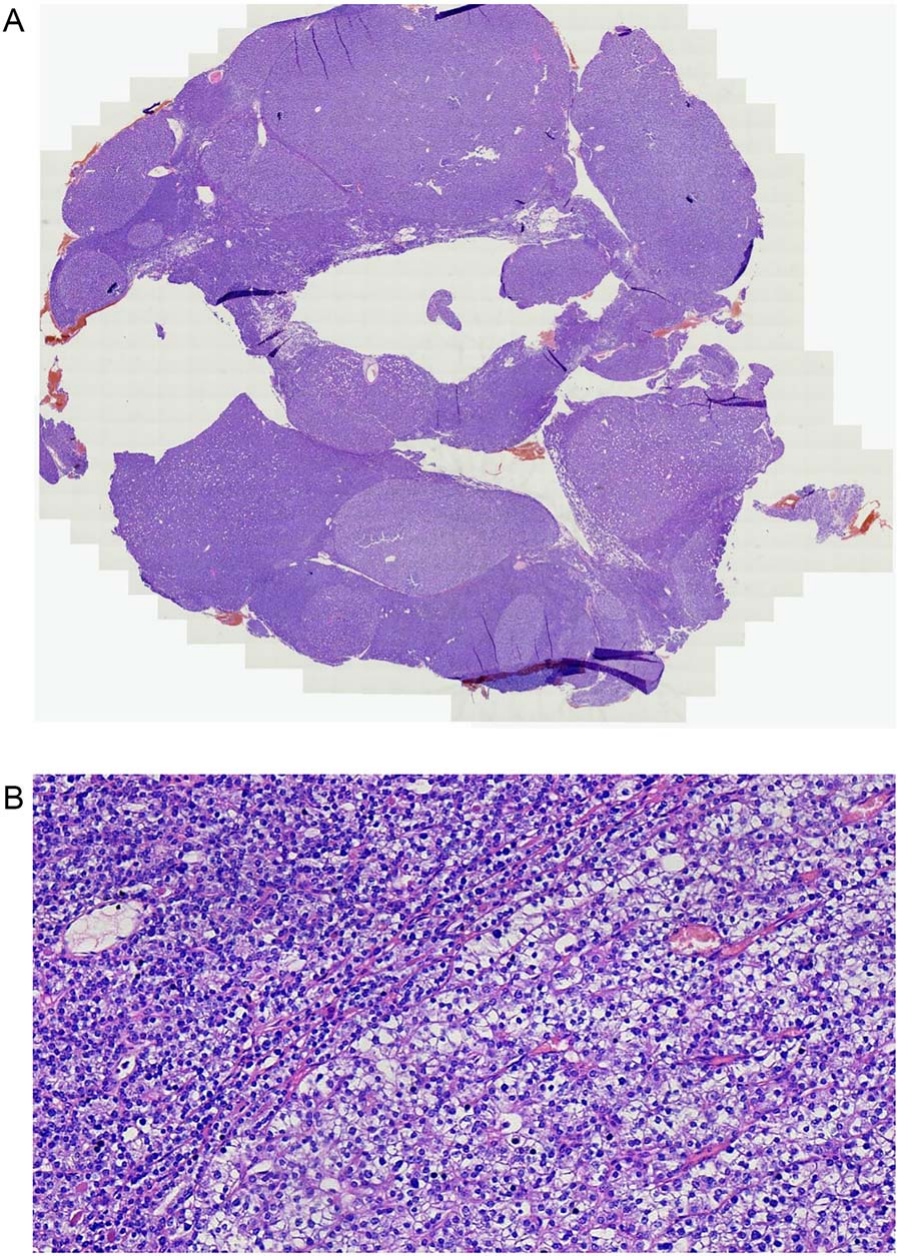
Resected parathyroid tissue showing a well-circumscribed adenoma composed of chief cells. (A) Low-power H&E section of resected parathyroid tissue showing a well-circumscribed lesion consistent with parathyroid adenoma. (B) High-power H&E section of resected parathyroid tissue showing chief-cell-predominant adenoma with preserved architecture and no evidence of atypia or capsular invasion.

After preoperative optimization with a single intravenous dose of zoledronic acid (4 mg) and intravenous hydration (4 L over 48 hours), she underwent focused parathyroidectomy with removal of enlarged left inferior and superior parathyroid adenomas, preserving the normal right-sided glands. This resulted in a marked fall in parathyroid hormone from 2300 pg/mL to 220 pg/mL after 24 hours, confirming biochemical cure. Histopathology confirmed a benign, chief cell–predominant parathyroid adenoma with no evidence of carcinoma (Fig. 8).

Following surgery, she developed severe hungry bone syndrome with symptomatic hypocalcemia (serum calcium 1.7 mmol/L), presenting with perioral paresthesias, carpopedal spasm, positive Chvostek and Trousseau signs. Management required high-dose intravenous calcium gluconate (2 g three times daily) alongside vitamin D supplementation, with gradual biochemical recovery over several weeks and weaning over two months. Clinically, she experienced significant pain reduction and functional recovery, and continues under endocrinology follow-up with planned orthopedic intervention for residual fractures.

## Discussion

Excess parathyroid hormone (PTH) enhances osteoclast-mediated bone resorption, leading to cortical bone loss, osteopenia, and increased risk of pathological fractures [4]. Hypercalcemia in primary hyperparathyroidism (PHPT) results from both enhanced bone resorption and increased renal tubular calcium reabsorption with concurrent phosphate wasting [5]. In regions with limited access to biochemical screening, PHPT often presents late with classical skeletal complications, including fragility fractures, and may closely mimic metastatic malignancy [6].

The diagnostic hallmark remains the demonstration of persistent hypercalcemia with inappropriately elevated PTH levels [7]. Imaging modalities, such as ultrasound or sestamibi scanning, assist in gland localization but cannot establish the diagnosis independently [8]. In our patient, the constellation of recurrent fractures, diffuse osteopenia, and markedly raised PTH confirmed PHPT after malignancy and myeloma were excluded.

Parathyroidectomy is the definitive treatment for symptomatic PHPT and is recommended even in certain asymptomatic patients who meet guideline criteria [9]. Medical therapy, including calcimimetics and antiresorptives, may serve as alternatives when surgery is contraindicated or as bridging strategies [9]. Our patient underwent parathyroidectomy, which was complicated by hungry bone syndrome, a well-recognized postoperative complication due to rapid remineralization of previously demineralized bone [10]. Surgical management was challenging in our setting, as profound bone fragility necessitated prioritization of parathyroidectomy before fracture fixation, limited access to intraoperative PTH monitoring required reliance on anatomic exploration, and the anticipated but severe hungry bone syndrome demanded prolonged and intensive calcium and vitamin D supplementation, reflecting the substantial skeletal calcium deficit from years of uncontrolled disease.

This case highlights a systems-level failure in early detection and highlights the need to consider metabolic bone disease in patients with unexplained pathological fractures after malignancy has been excluded, as timely access to basic biochemical screening and surgical intervention could enable earlier diagnosis, prevent unnecessary orthopedic and oncologic work-up, improve functional outcomes, and substantially reduce the associated clinical, social, and economic burden.

## Supplementary Material

Final_File_Submission_-_Authorship_Clarification_(OMCR-2025-804_omag023_R1)_omag023
