# Supplementary material for: Primary hyperparathyroidism presenting with pathological fractures mimicking malignancy: a case from Tanzania
Source: Oxf Med Case Reports. 2026 Mar 23;2026(3):omag023. doi: 10.1093/omcr/omag023 (PMC13007865; doi:10.1093/omcr/omag023)
Supplement: Final_File_Submission_-_Authorship_Clarification_(OMCR-2025-804_omag023_R1)_omag023 [file final_file_submission_-_authorship_clarification_(omcr-2025-804_omag023_r1)_omag023.docx]

 I am writing to formally request an update to the author list for our submitted case report.The proposed changes are as follows:

- Lutfi Abdallah has been moved to the position of second author.
- Dr. Hilda Makungu has been added as a new author.

These modifications reflect their substantive contributions to the clinical management and manuscript development of the case.

In line with the International Committee of Medical Journal Editors (ICMJE) authorship criteria, both authors have made significant contributions in critical revision of the manuscript for important intellectual content, and have approved the final version to be published. They agree to be accountable for all aspects of the work and to ensure the accuracy and integrity of the reported case.

Dr. Hilda Makungu’s contact details are provided below for your records:

- Email:  [hilda.makungu@gmail.com](mailto:hilda.makungu@gmail.com)
- ORCID ID: 0009-0004-5209-2741

All authors have been informed of and have agreed to these authorship changes. We respectfully request your approval to proceed with the revised author list and are happy to provide signed authorship or contributor statements if required.

Thank you for your consideration.

Kind regards,

Noorein Omar

Corresponding Author
